# Pathological findings in African buffaloes (*Syncerus caffer*) in South Africa

**DOI:** 10.4102/jsava.v92i0.2117

**Published:** 2021-08-17

**Authors:** Daniel B. Woodburn, Johan Steyl, Elizabeth C. du Plessis, Rick D. Last, Bjorn Reininghaus, Emily P. Mitchell

**Affiliations:** 1 Department of Pathobiology, Zoological Pathology Program, University of Illinois, Illinois, United States; 2 Department of Paraclinical Sciences and Centre for Wildlife Veterinary Studies, Faculty of Veterinary Science, University of Pretoria, Onderstepoort, South Africa; 3 IDEXX Laboratories, Johannesburg, South Africa; 4 Vetdiagnostix, Pietermaritzburg, South Africa; 5 Mpumulanga Veterinary Services, Nelspruit, South Africa; 6 National Zoological Garden, South African National Biodiversity Institute, Pretoria, South Africa

**Keywords:** African buffalo, *Syncerus caffer*, disease, pathology, wildlife veterinarians

## Abstract

The African buffalo (*Syncerus caffer*) is an iconic species of South African megafauna. As the farmed buffalo population expands, the potential impacts on population health and disease transmission warrant investigation. A retrospective study of skin biopsy and necropsy samples from 429 animals was performed to assess the spectrum of conditions seen in buffaloes in South Africa. Determination of the cause of death (or euthanasia) could not be made in 33.1% (136/411) of the necropsy cases submitted due to autolysis or the absence of significant lesions in the samples submitted. Infectious and parasitic diseases accounted for 53.5% (147/275) of adult fatal cases and non-infectious conditions accounted for 34.9% (96/275). Abortions and neonatal deaths made up 11.6% (32/275) of necropsy cases. Rift Valley fever, bovine viral diarrhoea, malignant catarrhal fever, tuberculosis, bacterial pneumonia, anaesthetic deaths, cachexia and hepatotoxic lesions were the most common causes of death. The range of infectious, parasitic and non-infectious diseases to which African buffaloes were susceptible was largely similar to diseases in domestic cattle which supports concerns regarding disease transmission between the two species. The similarity between diseases experienced in both species will assist wildlife veterinarians in the diagnosis and treatment of diseases in captive African buffaloes. The present study likely does not represent accurate disease prevalence data within the source population of buffaloes, and diseases such as anthrax, brucellosis and foot and mouth disease are under-represented in this study. Hepatic ductal plate abnormalities and haemorrhagic septicaemia have not, to our knowledge, been previously reported in African buffaloes.

## Introduction

African buffaloes (*Syncerus caffer*) are one of the most iconic species of South African megafauna and are included among the ‘Big Five’, animals prized by big-game hunters and ecotourists alike. As such, in recent decades there has been significant growth in the South African game farming industry in order to meet the increasing demand for buffaloes to stock game reserves and wildlife parks. Current population estimates range from 45 000 to 100 000 farmed buffaloes with an additional 40 000–50 000 free-ranging buffaloes within the Greater Kruger National Park Complex (KNP), Hluhluwe/iMfolosi Park (HiP) and Addo National Park (ANP, personal communication, J. Dippenaar).

As the farmed buffalo population expands, the potential impacts on population health and disease transmission are important. Standard farming practices may affect disease epidemiology via artificially increased population densities, intensive nutritional management, and increased contact between buffaloes, domestic cattle, and the human population. One example that highlights the potential for disease transmission is the outbreak of bovine tuberculosis (*Mycobacterium bovis*) in the free-ranging buffalo population in KNP. First diagnosed in KNP buffaloes in 1990 (Bengis et al. 1996), by 1998 the prevalence of tuberculosis in buffaloes had risen to 38.2% in the southernmost region of the park (De Vos et al. 2001; Rodwell et al. 2001). Bovine tuberculosis is now considered endemic within KNP and HiP, with infection documented in many other South African wildlife species, including a variety of ruminants, carnivores and primates (Michel et al. 2006). As members of the family Bovidae, African buffaloes are susceptible to many of the same economically important infectious diseases as domestic cattle (Caron et al. 2016; Michel & Bengis 2012). Most of the published literature on diseases in buffaloes therefore describe the epidemiology and seroprevalence of infectious diseases relevant to cattle including foot and mouth disease (Thomson & Bastos 2004; Wekesa et al. 2015); arboviruses (Kading et al. 2013); Rift Valley fever (Beechler et al. 2015; Jori et al. 2015); lumpy skin disease (Fagbo, Coetzer & Venter 2014); bovine viral diarrhoea virus (BVDV) (Kabongo & Van Vuuren 2004); malignant catarrhal fever (MCF) (Pfitzer et al. 2015); tuberculosis (Kalema-Zikusoka et al. 2005; Tavalire et al. 2018); non-tuberculous mycobacteria (Gcebe et al. 2013); brucellosis (Alexander et al. 2012; Gradwell et al. 1977; Herr & Marshall 1981; Motsi et al. 2013; Tanner et al. 2014); anthrax (Cossaboom et al. 2019; De Vos & Turnbull 2004; Ebedes 1976); leptospirosis (Atherstone, Picozzi & Kalema-Zikusoka 2014); Q-fever (Ndeereh et al. 2017) and theileriosis (Chaisi et al. 2011). Parasite surveys and case reports are also recorded including infestations with coccidia (Gorsich et al. 2014); giardia (Hogan et al. 2014); ixodid ticks (Moyo, Chakuya & Sungirai 2018); haemoparasites (Eygelaar et al. 2015; Gonçalves et al. 2018; Sisson et al. 2017); nematodes (Taylor, Skinner & Boomker 2013); sarcocysts (Dubey et al. 2014; Quant et al. 1997); sarcoptes (Munang’andu et al. 2010); demodex (Dräger & Paine 1980; Wolhuter et al. 2009); schistosomes (Beechler et al. 2017); taenia (Muma et al. 2014); and thelazia (Ayebazibwe et al. 2010). Very little information is present on non-infectious conditions (Lawrence, Foggin & Prozesky 2010). Vigilant disease surveillance and studies on the interface between such diseases in cattle and buffaloes are invaluable in protecting both animal and public health as well as food security (Ritz et al. 2013). If current and future disease surveillance programmes are to be successful, it is first necessary to fully understand the range of diseases to which buffaloes are susceptible. For these reasons, a retrospective study was performed to assess the spectrum of conditions seen in African buffaloes in South Africa, and to provide a frame of reference for veterinarians, pathologists, wildlife biologists, and managers of game reserves.

## Materials and methods

A retrospective review of buffalo cases submitted for pathological evaluation to four South African laboratories between 2001 and 2015 was performed. Gross necropsy and histopathology reports, as well as archival histopathology slides, were provided by the National Zoological Garden (NZG) (South African National Biodiversity Institute), the Faculty of Veterinary Science (FVS) at the University of Pretoria, Vet Diagnostix, and IDEXX Laboratories.

Each case was reviewed by a single pathologist (DW). Clinical history, sex, age, date of post-mortem examination, body condition, pathological findings and the results of ancillary tests were recorded where available. Estimated age was supplied by submitting veterinarians. All haematoxylin and eosin (H&E) stained slides as well as any accompanying special stains and immunohistochemical stains were reviewed, and morphologic diagnoses were assigned for each organ system. Each case was also assigned a final diagnosis to highlight the major disease process and cause of death or reason for euthanasia.

### Ethical considerations

This article followed all ethical standards for research without direct contact with human or animal subjects.

## Results

In total, archival materials from 429 individual African buffaloes were available for review. In 334 animals, sex was provided on submission: 178 (53.3%) females and 156 (46.7%) males. In 362 cases, an estimated age was provided: 190 (52.5%) adults, 51 (14.1%) subadults, 72 (19.9%) juveniles, 32 (8.8%) neonates and 17 (4.7%) foetuses. Many more animals were recorded as coming from game farms (*n* = 149, 50.3%) and national parks (*n* = 131, 44.3%) than from zoos (*n* = 16, 5.4%). As many cases had no information available regarding sex (*n* = 95, 22.1%), age (*n* = 67, 15.6%) and/or captivity status (*n* = 133, 31.0%), these characteristics were excluded from further analysis.

### Biopsy cases

Skin biopsies were submitted from 18 (6.1%, 18/429) buffaloes, two of which showed non-specific lymphoplasmacytic perivascular dermatitis with no identifiable cause. Intralesional bacteria, primarily cocci, were present in three cases with ulcerative and suppurative dermatitis. Skin biopsies from three buffaloes had segmental epidermal hyperplasia with associated parakeratosis, adnexal structure atrophy, and mild pigmentary incontinence with minimal associated dermal inflammation. Three cases of cutaneous fibropapilloma were characterised by exophytic masses composed of multiple frond-like projections lined by hyperplastic keratinised stratified squamous epithelium with variable orthokeratosis and parakeratosis. Epithelium was supported by dermal projections of densely collagenous fibrovascular stroma with many reactive fibroblasts. Demodex infestation was associated in proliferative, hyperkeratotic and granulomatous dermatitis (*n* = 1); while infestation with *Psoroptes* spp. was associated with proliferative and ulcerative dermatitis (*n* = 2). One lipid granuloma; two cases of focal necrotising ulcerative bacterial vulvar dermatitis with coccoid bacteria on the skin surface; one suspected endocrine dermatosis; and one case of leukoderma were diagnosed.

Determination of the cause of death (or euthanasia) could not be made in a large portion of the necropsy cases submitted (33.1%, 136/411). Failure to make a diagnosis was because of autolysis (6.3%, *n* = 26) or there being no life-threatening lesions in the tissues submitted (26.8%, *n* = 110). The cause of death for the remaining fatal cases (*n* = 275, 66.9%) is listed in Tables 1–4. Cause of death was based on the presence of life-threatening lesion/s in the tissues submitted, supported by ancillary clinical and microbiological data, where available. Where relevant, concurrent significant lesions are also reported.

**TABLE 1:** Cause of death in 275 African buffaloes: Abortion and neonatal death.

Category	Cause of death	Number	Proportion (%)
Abortion		19	6.9
	No cause determined	8	2.9
	Rift Valley fever virus infection	8	2.9
	Bovine viral diarrhoea virus infection	2	0.7
	*Staphylococcus lentus* infection	1	0.4
Neonatal death		13	4.7
	Congenital biliary atresia	5	1.8
	Dystocia	2	0.7
	Bovine viral diarrhoea virus infection	1	0.4
	Congenital hydrocephalus	1	0.4
	Traumatic injury	1	0.4
	Omphalophlebitis	1	0.4
	Septicaemia	1	0.4
	Nutritional myopathy	1	0.4

**Total**		**32**	**11.6**

### Abortions and neonatal deaths

Abortions and neonatal deaths accounted for 11.6% (32/275) of fatal cases. The number and proportion of cases of abortion or neonatal death are shown in Table 1.

**FIGURE 1:**
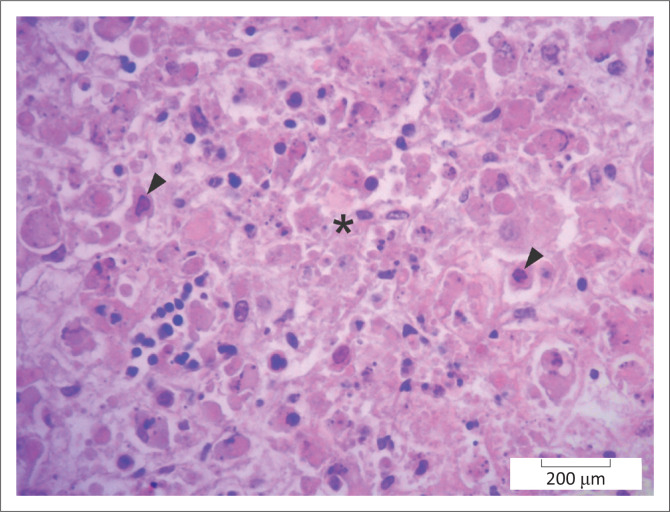
Liver, African buffalo. Area of hepatocellular necrosis (*) because of *Phlebovirus* infection (Rift Valley Fever). Several necrotic hepatocytes have intranuclear viral inclusions (arrowheads). Haematoxylin and eosin.

Of the total 32 cases, 19 (6.9%) cases were abortions. The cause of the abortions was not established in eight cases. Eight foetuses had lesions consistent with Rift valley fever (RVF) virus infection: severe multifocal random to massive necrotising hepatitis with (*n* = 7) or without (*n* = 1) intranuclear immunohistochemically positive viral inclusions (Figure 1). One case was positive on a polymerase chain reaction (PCR) test. Tissues from two foetuses tested positive on immunohistochemistry for BVDV and had cerebellar hypoplasia as well as lymph node and/or thymic lymphoid atrophy or hypoplasia. *Staphylocococcus lentus* was isolated from one case with intravascular cocci in the lung and kidney but no other lesions.

**FIGURE 2:**
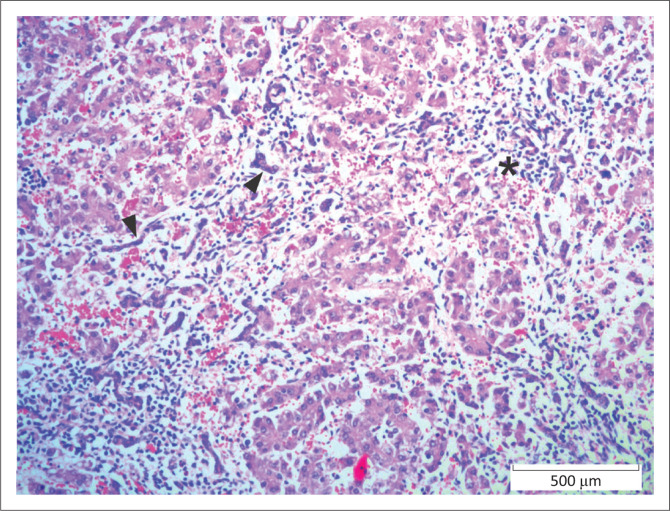
Liver, African buffalo. Hepatic ductal plate abnormality present at birth. Note the periportal lymphoplasmacytic inflammation (*) and multiple undeveloped bile ductules (arrowheads). Haematoxylin and eosin.

Of the 13 (4.7%) neonates submitted, five had congenital hepatic ductal plate malformation, one of which had concurrent bronchiolar dysplasia with associated pulmonary alveolar atelectasis and acute neutrophilic interstitial pneumonia; and another had concurrent pneumonia because of aspiration of milk. In these cases, there was marked variation in lobular size and poor organisation of hepatic cords. Portal triads had numerous small tortuous bile duct profiles that frequently extended into surrounding parenchyma and spanned adjacent portal areas and central veins (see Figure 2). Associated inflammation and fibrosis were minimal.

The death of the remaining neonates was attributed to dystocia (*n* = 2), BVDV infection (*n* = 1), congenital hydrocephalus (*n* = 1), traumatic injury (*n* = 1); necropurulent pleuritis and cystitis because of omphalophlebitis (*n* = 1); multifocal fibrinonecrotic hepatitis attributed to septicaemia (*n* = 1); and polyphasic myocardial and skeletal muscle necrosis suggestive of nutritional myopathy (*n* = 1).

### Infectious and parasitic diseases

Infectious and parasitic diseases accounted for 53.5% (147/275) of fatal cases. Table 2 shows the number and proportion of buffaloes that died because of infectious or parasitic diseases.

**TABLE 2:** Cause of death in 275 African buffaloes: Infectious and parasitic diseases.

Category	Cause of death	Number	Proportion (%)
Bacterial		106	38.5
	Bovine tuberculosis	55	20.0
	Bacterial pneumonia	13	4.7
	Clostridial infections	9	3.3
	Septicaemia	8	2.9
	Bacterial enterocolitis	7	2.5
	Abscesses	6	2.2
	Haemorrhagic septicaemia	6	2.2
	Traumatic thoracic infection	1	0.4
	Bacterial peritonitis	1	0.4
Viral		26	9.5
	Malignant catarrhal fever	15	3.5
	Viral encephalitis	4	1.5
	Viral enteritis	4	1.5
	Viral pneumonia	2	0.7
	Rabies	1	0.4
Fungal		3	1.1
	Rumenitis	2	0.7
	Abomasitis	1	0.4
Parasitic		12	4.4
	Theileriosis	7	2.5
	Coccidiosis	4	1.5
	Myocardial cestodiasis	1	0.4

**Total**		**147**	**53.5**

#### Bacterial

**FIGURE 3:**
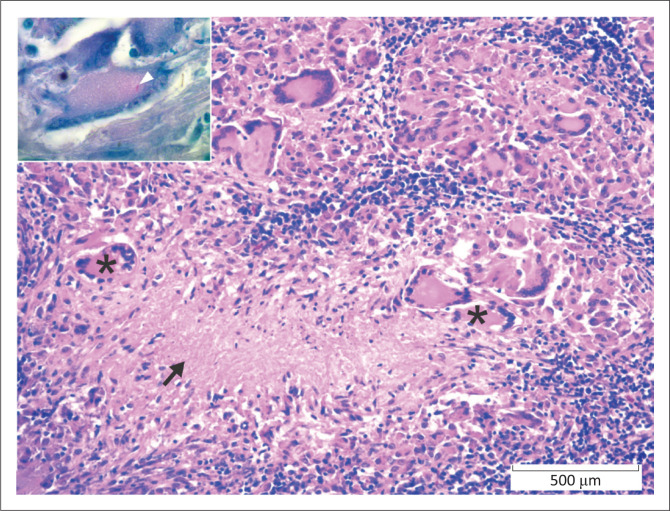
Lymph node, African buffalo. Granuloma with multinucleated giant cells (*) and central necrosis (arrow). Haematoxylin and eosin. *Inset*: A rare intracytoplasmic acid-fast (Ziehl-Neelsen) bacillus (arrowhead) is present within a giant cell. Ziehl Neelsen.

Bacterial conditions accounted for 38.5% (106/275) of fatal cases. The most common bacterial disease was tuberculosis, characteristic lesions of which were identified and detected in lymph nodes, lung and, rarely, in other tissues in 55 (20.0%) buffaloes. Lesions consisted of granulomas with multinucleated giant cells, central necrosis, peripheral lymphoplasmacytic inflammation, and in some cases mineralisation (Figure 3). Nineteen cases had small numbers of intra-histiocytic acid-fast bacilli morphologically compatible with *Mycobacterium* sp. (Ziehl Neelsen stain). In five of these cases, *M. bovis* infection was confirmed via PCR test. Scattered macrophage clusters in lung or lymph node were interpreted as early lesions in 17 cases.

Bacterial pneumonia and/or pleuropneumonia, with or without sepsis, was responsible for death in 13 (4.7%) adult buffaloes. *Mannheimia haemolytica* was isolated from one case. Marked multifocal subacute fibrinosuppurative to necrotising bronchopneumonia with unidentified coccobacilli was seen in three animals, one of which had concurrent theileriosis. One case had concurrent viral enteritis.

Confirmed or suspected clostridial infections were seen in nine (3.3%) buffaloes. Four buffaloes had necrotising suppurative myositis with variable numbers of plump spore-firming bacilli. Myositis was confirmed to be because of *C. septicum* (*n* = 1) and *C. chauvoei* (*n* = 1) by immunofluorescent antibody test; in two additional cases no further testing was performed. In five animals, lesions compatible with *Clostridium* spp. infection were seen: mild multifocal acute necrotising myocarditis with thrombosis (*n* = 1); necrohaemorrhagic enteritis (*n* = 1); and severe necrohaemorrhagic abomasitis, with intra-lesional large bacterial rods (*n* = 3). Cerebellum Purkinje cell necrosis, consistent with intoxication with *Solanum kwebense,* was also present in one of the latter cases.

In eight (2.9%) buffaloes, death was judged to be because of septicaemia, without identification of the pathogens responsible. Lesions included moderate multifocal random acute necrotising lesions in the liver (*n* = 4), lung (*n* = 3), spleen (*n* = 2), hepatic portal triads (*n* = 1), pleura (*n* = 1), myocardium and heart valves (*n* = 1). In one case, septicaemia was associated with severe chronic fibrinosuppurative pleuritis with intralesional coccobacilli (associated with a penetrating foreign body from the reticulum); in another the source of the septicaemia was valvular endocarditis.

Seven (2.5%) buffaloes died of complications of confirmed or suspected bacterial enterocolitis, in which there was necrosis and ulceration of the intestinal mucosa accompanied by predominately fibrinopurulent inflammation. From one animal with concurrent peritonitis, *Escherichia coli* was isolated; in another a *Salmonella* spp. was cultured. Samples were not submitted for culture in other animals. Mild segmental acute necrotising suppurative enteritis had intralesional coccobacilli (*n* = 1); severe multifocal acute ulcerative enteritis with mixed bacteria (*n* = 1); and mild focal acute ulcerative colitis with intralesional bacilli (*n* = 1) were reported. Bacterial disease was suspected because of mild diffuse acute neutrophilic enteritis in one animal; and to haemorrhagic enteritis and jejunal volvulus in another.

In six (2.2%) buffaloes, death was attributed to the presence of abscesses. One of two buffaloes with brain abscesses also had rumen acidosis. *Trueperella pyogenes* was isolated (*n* = 1) and intralesional filamentous bacteria consistent with *Fusobacterium necrophorum* (*n* = 1) were present in buffaloes with abscesses in multiple tissues. One animal each had *T. pyogenes* abscesses in the mandible and renal pelvis.

Five (1.8%) buffaloes died because of haemorrhagic septicaemia caused by *Pasteurella multocida.* Affected animals had variable combinations of multifocal necrohaemorrhagic or fibrinopurulent bronchopneumonia and pleuritis (*n* = 2); pulmonary haemorrhage (*n* = 1), epicarditis (*n* = 1), hepatitis (*n* = 1), lymphadenitis (*n* = 3), tonsillitis (*n* = 2), lymphangitis (*n* = 1), nephritis (*n* = 1), myositis (*n* = 1), thymitis (*n* = 1) and adrenocortical haemorrhage (*n* = 1) with small numbers of intravascular and/or intralesional coccobacilli. One additional buffalo with severe multifocal to coalescing subacute necropurulent pharyngeal myositis was presumed to also have had haemorrhagic septicaemia.

One buffalo died of severe chronic fibrinosuppurative epicarditis attributed to traumatic inoculation of bacteria as a result of foreign body migration from the reticulum. One animal died as a result of marked acute fibrinosuppurative peritonitis with intralesional coccobacilli.

#### Viral

**FIGURE 4:**
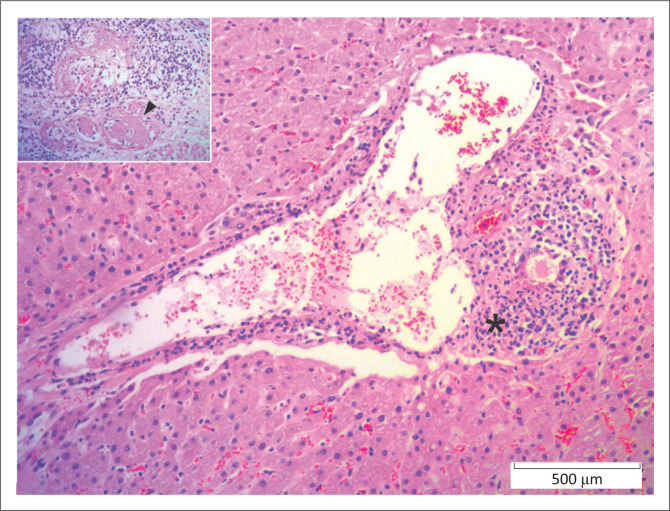
Liver, African buffalo. Portal triad with necrotising and lymphocytic vasculitis (*) because of *Ovine herpes virus*-2 infection (malignant catarrhal fever). *Inset*: vascular profiles are obscured by fibrinoid necrosis (arrowhead). Haematoxylin and eosin.

Viral diseases accounted for 9.5% (26/275) of fatal cases. Fifteen individuals (3.5%) had lesions consistent with MCF. Necrotising vasculitis with fibrinoid change and associated mural and perivascular lymphoplasmacytic inflammation were most common in the liver (*n* = 10), kidney (*n* = 9) and lung (*n* = 9), but were also seen in the intestine (*n* = 5), heart (*n* = 5), spleen (*n* = 3), lymph node (*n* = 2), rumen (*n* = 2), brain (*n* = 2) and retrobulbar adipose tissue (*n* = 1) (Figure 4). In seven of these cases, ancillary PCR testing performed at the time of necropsy confirmed Ovine Herpes Virus 2 (OvHV-2, *n* = 3) or Alcelaphine Herpesvirus-1 (AlHV-1, *n* = 4)

Viral encephalitis was diagnosed in four (1.5%) buffaloes as a result of a history of ataxia, recumbency and acute death and the presence of spinal cord glial apoptosis, neuronal degeneration and/or cerebral oedema. Four (1.5%) animals died of enteric lesions consistent with viral infection. Three of these buffaloes had crypt epithelial necrosis, while the remaining only had residual intestinal villus blunting and fusion, and was cachexic.

Two (0.7%) buffaloes were thought to have died of viral pneumonia because of the presence of severe multifocal to coalescing acute serofibrinous interstitial pneumonia with epithelial syncytia. However, neither virus isolation nor PCR test were performed. One affected animal also had ruminal acidosis. One animal died of confirmed rabies infection.

#### Fungal

Fungal diseases accounted for 1.1% (3/275) of fatal cases. Two buffaloes had proliferative and ulcerative fungal rumenitis; and in another zygomycete fungal ulcerative abomasitis resulted in perforation and peritonitis.

#### Parasitic

**FIGURE 5:**
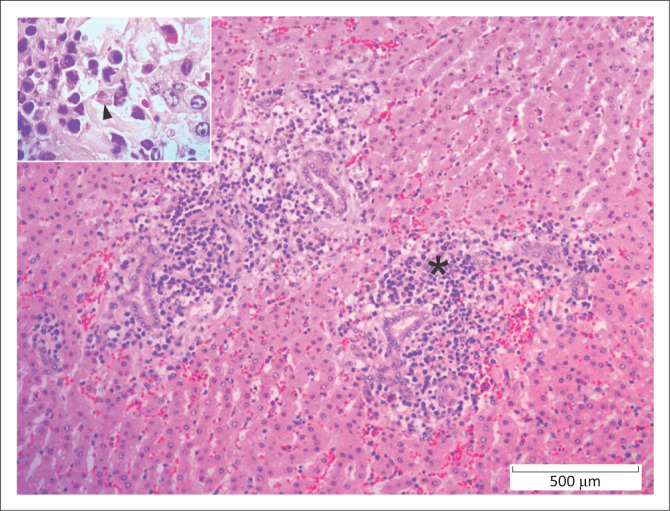
Liver, African buffalo. Lymphoplasmacytic portal hepatitis (*) because of *Theileriosis* infection. *Inset*. Lymphoblastic cell with intracytoplasmic schizonts (arrowhead). Haematoxylin and eosin.

Parasitic diseases accounted for 4.4% (12/275) of fatal cases. Theileriosis (Figure 5) was diagnosed in seven (2.5%) buffaloes with widespread lymphoplasmacytic infiltrates, including atypical blastic cells with or without protozoa, in the hepatic portal area (*n* = 5), lymph node (*n* = 4), spleen (*n* = 3), renal interstitium (*n* = 3), myocardium (*n* = 2) and/or pulmonary interstitium (*n* = 1). Only one case was confirmed by PCR test. Additional lesions included marked subacute fibrinosuppurative bronchopneumonia with few fibrin thrombi (*n* = 1), marked segmental acute fibrinonecrotic and ulcerative abomasitis (*n* = 1), marked focal subacute necrotising nephritis (*n* = 1) and severe subacute fibrinonecrotic lymphadenitis (*n* = 1).

Four (1.5%) buffaloes died of severe infestation of the intestine and diarrhoea associated with coccidiosis. Coccidia was most commonly present in the small intestine, and associated with mild often eosinophilic inflammation. Coccidian burden varied considerably between individuals and between different intestinal sections of the same individual. In addition, mild intestinal epithelial infestation with coccidia was present in 10 (3.6%) other animals. No other significant lesions in the tissues submitted were present in two of these cases; and one animal each had concurrent compressive myelopathy, displaced abomasum, end stage kidney disease, haemochromatosis, pneumonia as a result of aspiration of rumen fluid and cachexia.

Multiple encysted larval cestodes were associated with mild granulomatous myocarditis in one buffalo.

### Non-infectious causes of death

Non-infectious conditions accounted for 34.8% (96/276) of fatal cases. The number and proportion of non-infectious causes of death is listed in Table 3. Death during or shortly after anaesthesia was seen in 20 (7.2%) buffaloes. Lesions seen included acute renal tubular necrosis (*n* = 4), pulmonary congestion and/or oedema (*n* = 4), myocardial oedema (*n* = 1), pulmonary atelectasis (*n* = 1), intact erythrocytes in lymph node sinuses (interpreted as drainage from areas of haemorrhage, *n* = 1), acute myocardial necrosis (*n* = 1), perivascular cerebral haemorrhage (*n* = 1), tracheal haemorrhage (*n* = 1), pneumonia as a result of aspiration of rumen fluid (*n* = 2), centrilobular liver necrosis (*n* = 1), focal neutrophilic necrotising haemorrhagic myocarditis (*n* = 1), hypoxic encephalopathy with neuronal necrosis (*n* = 1) and cerebral oedema (*n* = 1). One affected animal was cachexic with endoparasites, anaemia and sepsis. Three animals were diagnosed with anaesthetic-induced hyperthermia as a result of hepatocellular degeneration (*n* = 3), acute renal tubular necrosis (*n* = 2), multifocal fibrin thrombi (*n* = 1) and multifocal acute cerebral neuronal necrosis (*n* = 2). In four animals, no lesions were seen in the tissues submitted.

**TABLE 3:** Cause of death in 275 African buffaloes: Non-infectious diseases.

Cause of death	Number	Proportion (%)
Anaesthetic related	20	7.2
Cachexia	19	6.9
Hepatotoxicity	11	4.0
Myocardial necrosis	10	3.6
Capture myopathy	9	3.3
Trauma	5	1.8
Rumen acidosis	4	1.4
Aspiration pneumonia	2	0.7
Salt and microcystin toxicity	2	0.7
Subacute meningoencephalitis	2	0.7
Oesophageal phytobezoar	1	0.4
Clostantel toxicity	1	0.4
Drowning	1	0.4
End stage kidney disease	1	0.4
Haemorrhagic enteritis	1	0.4
Pressure myopathy	1	0.4
Tick toxicosis	1	0.4
Multicentric lymphoma	1	0.4
Acute nephrosis	1	0.4
Cerebrocortical laminar necrosis	1	0.4
Pregnancy toxaemia	1	0.4
Acute liver necrosis	1	0.4

**Total**	**96**	**34.8**

Nineteen buffaloes (6.9%) died of malnutrition/starvation with lesions including skeletal muscle atrophy, hepatocellular atrophy with or without concomitant lipidosis, serous atrophy of peri-renal and epicardial fat, and pancreatic zymogen granule depletion. In six animals, no lesions were seen in the tissues submitted. In the remainder, the relationship between various mild lesions and death because of cachexia was uncertain: multifocal proliferative glomerulopathy; multifocal renal interstitial fibrosis; multifocal cardiomyocyte degeneration and megakaryosis, multifocal pulmonary arteriolar mineralisation, aortic arteriosclerosis, cerebral gliosis or oedema, cutaneous lice infestation, paramphistome infestation of the rumen, coccidiosis, enteritis, goitre and pneumonia as a result of aspiration of rumen fluid.

Eleven (4.0%) buffaloes died because of hepatotoxicity. Acute disease (*n* = 5) was characterised by centrilobular hepatocyte necrosis and loss. One affected animal also had terminal pneumonia as a result of aspiration of rumen fluid and mild acute myocardial necrosis. Chronic cases (*n* = 6) had additional changes including lobular collapse, hepatocellular megakaryosis, biliary hyperplasia, and bridging fibrosis. Two of these cases also had aspiration pneumonia; and one was suffering from multicentric lymphoma. Similar lesions were seen in an additional animal that was also cachexic. Given the paucity of historical information for most cases, a specific cause could not be determined for any case.

Ten (3.6%) buffaloes died as a result of multiphasic subacute to chronic multifocal myocardial necrosis with or without myofibre atrophy (*n* = 3), myoglobinuria (*n* = 1) and chronic pulmonary oedema (*n* = 1). These lesions were interpreted as nutritional or toxic myopathy. One had fibrinoid vascular necrosis attributed to concurrent copper deficiency although no tissue mineral testing was performed. Myocardial fibrosis in one buffalo was interpreted as chronic capture or nutritional myopathy. Two animals had concurrent marked diffuse epidermal hyperplasia and ortho/parakeratosis with secondary bacterial and fungal dermatitis in addition to the myocardial lesions.

Nine (3.3%) animals were diagnosed with capture myopathy because of history of recent anaesthesia and/or translocation and the presence of acute skeletal muscle degeneration and/or necrosis. Additional lesions seen in these cases included acute myocardial necrosis (*n* = 5), myoglobinuric nephrosis (*n* = 2), centrilobular hepatic necrosis (*n* = 2) and pulmonary congestion and oedema (*n* = 1). One animal had acute myocardial necrosis and subacute skeletal muscle necrosis. One affected animal also had lesions consistent with MCF.

Five (1.8%) animals died or were euthanised as a result of traumatic injury: a fractured foot, gangrenous wound, and two by predation. In one case, the trauma was not specified.

Four (1.4%) buffaloes died of rumen acidosis segmental ruminal epithelial hyperplasia and parakeratosis, with multifocal areas of epithelial necrosis, microabscesses and ulceration and/or mucosal oedema. One also had ruminal infestation with paramphistomes; and another with suspected viral pneumonia. Two animals with ruminal ulceration or rumenitis also had secondary embolic pneumonia. An additional four animals had similar mild ruminal lesions, but died of other causes: brain abscess (*n* = 1), haemorrhagic septicaemia (*n* = 1), anaesthetic death (*n* = 1), cachexia (*n* = 1),

Two (0.7%) animals died of aspiration pneumonia as a result of aspiration of activated charcoal (*n* = 1) and a foreign body (*n* = 1), respectively. Two (0.7%) died of cerebral oedema, acute laminar cortical necrosis and cerebral perivascular haemorrhage; as well as periacinar hepatocyte necrosis and skeletal muscle necrosis. The diagnosis was a combination of water deprivation and blue-green algal toxicity.

Two buffaloes (0.7%) died of moderate multifocal lymphoplasmacytic meningoencephalitis of unknown aetiology. One of these animals had significant numbers of macrophages in the inflammatory response; in the other, inflammation was necrotising and concurrent pneumonia as a result of aspiration of rumen fluid was present.

The following single cases of death or euthanasia were seen: because of choke, bloat and pulmonary congestion and oedema as a result of oesophageal phytobezoar; presumed closantel toxicity with marked cerebral white matter spongiosis, mild neuronal degeneration and gliosis, perivascular cerebral oedema and skeletal muscle degeneration; drowning; a combination of end stage kidney disease and haemochromatosis; pressure myopathy; rhabdomyolysis attributed to tick toxicosis; multicentric small cell lymphoma; acute nephrosis the cause of which could not be determined; marked acute cerebrocortical laminar necrosis; pregnancy toxaemia; acute liver necrosis of unknown cause.

### Miscellaneous non-fatal lesions, not the cause of death

In addition to the cause of death in few cases miscellaneous lesions were also present. These were however deemed of no specific clinical significance. The number and proportion of cases with miscellaneous lesions are listed in Table 4. The proportion of cases is based on the number of animals for which the relevant tissue was submitted.

**TABLE 4:** Miscellaneous non-fatal lesions in African buffaloes.

Cause of death	Number	Proportion (%)
Anaesthetic related	20	4.7
Cachexia	19	4.4
Hepatotoxicity	11	2.6
Capture myopathy	9	2.1
Myocardial necrosis	7	1.6
Rumen acidosis	7	1.6
Trauma	5	1.2
Aspiration pneumonia	2	0.5
Salt and microcystin toxicity	2	0.5
Subacute meningoencephalitis	2	0.5
Oesophageal phytobezoar	1	0.2
Clostantel toxicity	1	0.2
Drowning	1	0.2
End stage kidney disease	1	0.2
Haemorrhagic enteritis	1	0.2
Pressure myopathy	1	0.2
Tick toxicosis	1	0.2
Multicentric lymphoma	1	0.2
Acute nephrosis	1	0.2
Cerebrocortical laminar necrosis	1	0.2
Pregnancy toxaemia	1	0.2
Acute liver necrosis	1	0.2

Lymph node reactive change was noted in 154 (62.6%, 154/202) individuals for which lymph nodes were submitted: lymphoid follicular hyperplasia, sinus histiocytosis and/or plasmacytosis, and mild lymph node oedema. In 35 (14.2%) cases, lymph node changes consistent with draining areas of haemorrhage were present, including flooding of medullary sinuses with erythrocytes and proteinaceous fluid. Additionally, there were variable numbers of sinus histiocytes with intracytoplasmic erythrocyte fragments and/or dark brown granular hemosiderin pigment. Eight (3.3%) adult individuals had lymphoid depletion noted in the lymph nodes with a paucity of lymphocytes and follicular structures. One animal had pyogranulomatous lymphadenitis with intralesional bacilli (not consistent with tuberculosis); and another buffalo had generalised neutrophilic lymphadenitis of which a cause could not be determined.

In addition, 44 (18.0%, 44/244) buffaloes had varying degrees of splenic lymphoid follicular hyperplasia and sinus histiocytosis and/or plasmacytosis consistent with reactive change. Reactive change was considered an incidental finding resulting from antigenic stimulation because of a variety of causes. In 43 (17.6%) individuals, the splenic red pulp contained increased numbers of hemosiderin-laden macrophages. Six (2.5%) adult animals had splenic lymphoid atrophy, in which there were both decreased numbers of lymphoid follicles and a paucity of lymphocytes within follicles.

Fourteen (60.9%, 14/23) animals had ruminal epithelial mineralisation. Superficial ruminal epithelial cells were deeply basophilic, stippled, and fragmented. In some cases, mineralisation was accompanied by mild epithelial hyperplasia. In two (8.7%, 2/23) cases, the rumen was infested with paramphistomes.

One animal had focal neutrophilic and eosinophilic abomasitis, which was likely as a result of internal parasites. Enterocolitis was present in 14 (27.5%, 14/51) buffaloes with no apparent specific aetiology. Increased numbers of lymphocytes, plasma cells, and eosinophils in varying proportions infiltrated the lamina propria of the small and/or large intestines with minimal mucosal damage.

The most prevalent hepatic lesion, noted in 42 (15.0%, 42/280) animals, was mild lymphoplasmacytic periportal hepatitis with mild biliary hyperplasia and periportal fibrosis. Hepatic haemochromatosis was present in eight (2.9%) animals. One buffalo had hepatic infestation with *Fasciola* spp.

Thirty two (10.4%, 32/309) buffaloes had small perivascular and peri-bronchial clusters of macrophages with abundant dark brown to black, granular, sometimes refractile, intracytoplasmic pigment. In some individuals, similar pigment-laden macrophages were also noted in the medullae of tracheobronchial lymph nodes. Pigment was consistent with carbon and/or silica and accumulation within macrophages was likely secondary to chronic inhalation of particulate matter (anthracosis). One animal each had mild eosinophilic pneumonia; and mild lymphohistiocytic pneumonia.

Twenty five (9.5%, 25/264) buffaloes had mild chronic interstitial nephritis, characterised by multifocal interstitial infiltrates of lymphocytes, plasma cells, occasional macrophages, and variable amounts of accompanying fibrosis. Eleven (4.2%) animals had small numbers of renal tubules, predominately within the medulla, with mineralised foci in their lumina. Seven (2.7%) individuals had membranous glomerulonephritis characterised by mild to moderate thickening of capillary basement membranes and Bowman’s capsules as well as segmental to global increases in the amount of mesangial matrix.

Ten (71.4%, 10/14) skin samples from necropsy cases had dermal inflammation but no detectable etiologic agents. Inflammation ranged from neutrophilic to lymphoplasmacytic, and in one case granulomatous, and there were variable degrees of associated dermal fibrosis and epidermal hyperplasia.

Six (2.3%, 6/265) individuals had small focal to multifocal areas of myocardial and/or endocardial mineralisation that were likely related to previous resolved injury. One animal had mild localised lymphocytic and neutrophilic vasculitis.

Five (12.3%, 5/38) animals had adrenocortical hyperplasia. Four buffaloes (16%, 4/25) had mild hyperplasia of the thyroid gland (hyperplastic goitre). Two (2%, 2/100) had skeletal muscle cysts compatible with *Sarcocystis cafferi* in skeletal muscle (adjacent to the trachea). One animal each had cerebral oedema; and spinal cord axonal degeneration. No lesions were noted in the reproductive system in the 14 cases for which reproductive tissue was available.

## Discussion

Based on these findings, the range of infectious, parasitic and non-infectious diseases to which African buffaloes are susceptible is largely similar to diseases in domestic cattle which supports concerns regarding disease transmission between the two species. The similarity between diseases experienced in both species may assist wildlife veterinarians in the diagnosis and treatment of diseases in captive African buffaloes. The histological appearance of *Mannheimia haemolytica* associated pneumonia, *Clostridium* myositis and abomasitis, *E. coli* and *Salmonella* enteritis, *Trueperella pyogenes* and *Fusobacterium necrophorum* infections, rabies were similar to those described in cattle (eds. Coetzer, Thomson & Tustin 2004).

In addition to those diseases known to be transmitted from buffaloes to cattle, some diseases were likely transmitted to buffaloes from cattle. The presence of BVD in buffalo foetuses likely reflects infection from cattle, as the virus has not been detected in buffaloes from the KNP (Kabongo & Van Vuuren 2004). Similarly, skin lesions because of bovine papilloma virus, as seen in these buffaloes, are recorded in several wild ruminants, including buffaloes (Williams et al. 2011). However, testing for viral genetic material was not performed so this diagnosis could not be confirmed.

While the source of viral encephalitis was not determined in the buffaloes in this series, the lesions resembled those in domestic horses in South Africa as a result of West Nile virus infection (Williams 2014). Serological evidence of alpha and flavivirus infections is recorded in various African wild animals (Kading et al. 2013). Many species of domestic and wild animals may therefore be susceptible to disease because of these infections, particularly when climate conditions favour the proliferation of insect vectors and/or host immune suppression (e.g. late winter especially during periods of drought). Similarly, the presence of buffalo abortions because of RVF, as well as cases of infestation with *Fasciola* spp. and paramphistomes, are suggestive that both cattle and buffaloes may be affected by diseases from a common source. Cases of MCF were similar to those described previously (Pfitzer et al. 2015). The OHV-2 infection likely indicate the infection of buffaloes from domestic ruminants; while cases because of AlHV-1 emphasise the need to manage contact between wild ruminant species on game farms.

The appearance of tuberculous lesions and those of theileriosis corresponded to previous reports in buffaloes (Bengis et al. 1996; Clift et al. 2020; De Vos et al. 2001; Laisse et al. 2011). Sarcocyst morphology was consistent with *Sarcocystis cafferi* as previously reported in African buffaloes (Dubey et al. 2014; Quant et al. 1997) and occurred only in free-ranging buffaloes presumably because, in such animals, predators are present to complete the indirect life cycle.

Some cattle diseases reported here have not, to our knowledge, been previously reported in African buffaloes. Hepatic ductal plate abnormalities are uncommon in cattle and typically associated with more fibrosis than was present in these cases (Bourque et al. 2001; Yoshikawa et al. 2002). A genetic predisposition is more likely than a toxic aetiology, as toxic hepatic ductal plate abnormalities are not seen in other ruminants in South Africa. Haemorrhagic septicaemia, with similar pathology to that described in African buffaloes in this study, is common in water buffaloes (*Bubalis bubalis*) (Praveena et al. 2014) suggesting that Bovidae share a predisposition to *Pasteurella multocida* infection. *Staphylococcus lentus* is part of a coagulase negative antibiotic resistant *Staphylococcus sciuri* group that is widespread in nature, and principally causes wound infections and mastitis in animals (Stepanovic et al. 2001). Its role in the abortion of the single buffalo in this series is uncertain.

Given the importance of infectious disease in buffaloes, it is not surprising that non-infectious disease is less frequently reported. The pathology of deaths that occur during or shortly after anaesthesia has not, to our knowledge, been documented in African buffaloes. Cardiorespiratory, renal and central nervous system changes were similar to those described in wildlife in general (Meltzer & Kock 2012). Similarly, myocardial and skeletal muscle lesions were characteristic of capture myopathy (Breed et al. 2019). The presence of subacute muscle lesions in animals dying with acute capture myopathy could be because of prior immobilisation or concurrent nutritional myopathy. Hepatic haemochromatosis is rare in domestic cattle, although cases are described in the Saler breed (O’Toole et al. 2001). The cause in buffaloes is unknown.

Various conditions, often seen in cattle being intensively managed, were also seen in buffaloes. Coccidian speciation was not performed, though *Eimeria sp.* infection has previously been reported in buffaloes. The prevalence and intensity of coccidian infestation in free-ranging buffaloes is affected by season (Gorsich et al. 2014). Wildlife veterinarians may need to examine factors such as animal density, water drainage into and faecal contamination of feeding areas in farmed buffaloes, which may facilitate occasional fatal coccidiosis.

Some conditions likely had a nutritional aetiology, as a result of artificial nutrition and/or drought conditions. Lesions consistent with acidosis likely explain the cases of fungal rumenitis and brain abscesses. Given the presence of lesions characteristic of nutritional myopathy, and zinc-responsive dermatosis, supplementation of multiple minerals should be considered, particularly if buffaloes are fed irrigated lucerne (Mbatha et al. 2012). The presence of nutritional myopathy in a neonate is suggestive of significant underlying nutritional deficiency disease in adult buffalo cows. Since free-ranging buffaloes do not normally suffer from blue-green algae toxicity (Bengis et al. 2016) because of their habit of wading into water bodies to drink, the water deprivation and blue-green algae toxicity cases were likely because of accidental temporary suspension and poor hygiene of water supply in farmed animals.

In addition, myocardial (Lawrence et al. 2010) as well as hepatic and neurological lesions characteristic of plant toxicities in cattle in South Africa (Kellerman et al. 2005) indicate that, as in cattle, buffaloes with insufficient alternative good quality feed may ingest toxic plants. Ruminal epithelial hyperplasia and mineralisation are reported in impala (*Aepyceros melampus*). The lesion is seen most commonly in the dry season and at locations at lower altitude where forage protein is low and dietary fibre and browse content is high, and may therefore be a result of a diet rich in tannins (Lane 1995). How this relates to the same lesion in buffaloes is unknown, as they are typically grazers. More research is needed on how artificial diets in captivity may affect buffalo health.

As a retrospective analysis spanning 15 years, the present study likely does not represent accurate disease prevalence data within the source population of buffaloes because of several factors. Firstly, specimens were not collected in a systematic manner. For example, reproductive tract tissue was rarely submitted, making detailed evaluation of this organ system impossible. A definitive diagnosis of abortion is most likely if foetal and placental tissues are promptly submitted to a diagnostic lab; yet placenta was rarely submitted. The proportion of abortions in which a diagnosis could be made in these buffaloes is similar to that described in cattle (Wolf-Jäckel et al. 2020).

In addition, clinical data was rarely detailed; and additional tests were often not possible because of failure to submit the relevant samples, sample post-mortem change or financial considerations. For example, had the clinical history of one bull with myoglobinuria included that it had been observed aggressively fighting with other males, the cause of muscle necrosis and subsequent pigmentary nephrosis could have been confirmed as exertional rhabdomyolysis. Similarly, without more extensive clinical history, the cause of cachexia could rarely be determined. The value of the study would have been greatly improved if such information was submitted with the tissues for analysis and a full set of tissues collected from all animals. Lastly, many tissues had significant autolytic change which hindered meaningful histological interpretation and diagnosis.

Some diseases to which buffaloes are susceptible were under-represented in this study. Diseases such as anthrax, brucellosis and foot and mouth disease (FMD) are endemic in buffaloes in KNP and other locations (Cossaboom et al. 2019; De Vos & Turnbull 2004; Gradwell et al. 1977; Herr & Marshall 1981; Motsi et al. 2013; Vosloo et al. 2009) and are diagnosed by experienced field staff without submitting samples for pathological examination. In addition, the transport of samples out of FMD endemic areas to regional laboratories is restricted. Ticks, internal parasites, haemoparasites and *Bartonella* spp. (Gonçalves et al. 2018) were likely similarly under-represented as blood or cytology samples were rarely submitted.

In contrast, the high prevalence of tuberculosis in this series is likely as a result of targeted surveillance for the disease in free-ranging and captive animals and the requirement to confirm the diagnosis by histological examination. Diagnosis is rarely confirmed by culture and/or PCR in free-ranging buffaloes from the endemic regions of South Africa, because of cost considerations and the ubiquitous presence of *M. bovis* in the buffalo population there. Farmed buffaloes that test positive for tuberculosis and are euthanised to confirm or rule out the infection for disease control purposes (Musoke et al. 2015).

This study confirmed that African buffaloes are susceptible to many of the same diseases as domestic cattle in South Africa. Therefore, in the absence of definitive tests, practitioners should assume that cattle and buffaloes share pathogens where they co-exist and that diseases in buffaloes may present similarly to those in cattle. As such, it is important to recognise that buffaloes may play a pivotal role in disease epidemiology with significant impacts on food security, public health, and wildlife conservation. For these reasons, continued and diligent surveillance of captive, farmed, and free-ranging populations alike is imperative.
